# Dietary Fatty Acids and Immune Response to Food-Borne Bacterial Infections

**DOI:** 10.3390/nu5051801

**Published:** 2013-05-22

**Authors:** Lisa M. Harrison, Kannan V. Balan, Uma S. Babu

**Affiliations:** Office of Applied Research and Safety Assessment, Center for Food Safety and Applied Nutrition, Food and Drug Administration, 8301 Muirkirk Road, Laurel, MD 20708, USA; E-Mails: kannan.balan@fda.hhs.gov (K.V.B.); uma.babu@fda.hhs.gov (U.S.B.)

**Keywords:** fatty acids, immune response, food-borne, infection

## Abstract

Functional innate and acquired immune responses are required to protect the host from pathogenic bacterial infections. Modulation of host immune functions may have beneficial or deleterious effects on disease outcome. Different types of dietary fatty acids have been shown to have variable effects on bacterial clearance and disease outcome through suppression or activation of immune responses. Therefore, we have chosen to review research across experimental models and food sources on the effects of commonly consumed fatty acids on the most common food-borne pathogens, including *Salmonella* sp., *Campylobacter* sp., Shiga toxin-producing *Escherichia coli*, *Shigella* sp., *Listeria monocytogenes*, and *Staphylococcus aureus*. Altogether, the compilation of literature suggests that no single fatty acid is an answer for protection from all food-borne pathogens, and further research is necessary to determine the best approach to improve disease outcomes.

## 1. Introduction

There are two main branches of the immune system, namely innate and acquired immunity. Cells associated with innate immunity offer the first line of defense upon exposure to foreign invaders. Innate immunity is also known as the non-specific immune system, which is the first line of defense against infections. This does not require previous exposure to an antigen and it includes barriers such as skin and mucous membranes, phagocytic cells such as macrophages, polymorphonuclear leukocytes (PMN), complement system, antimicrobial substances and other inflammatory cells. Acquired or specific immunity on the other hand results in the recognition of antigens from previous exposures by developing cellular memory. Acquired immunity is provided mainly by two types of lymphocytes namely the T and B lymphocytes, which recognize antigens via specific receptors. The immunity offered by B cells and their antibodies is referred to as the humoral response. T lymphocytes are responsible for the cell-mediated immune response, which is mediated by a variety of cytokines and soluble factors that ultimately help eradicate the invading pathogen. Host defense against microbial pathogens involves coordination of multiple signals between cells of both the innate and acquired immune systems. The cascade of events includes recruiting macrophages and neutrophils to the site of infection, releasing antimicrobial effectors and induction of the acquired immune response, which will ultimately result in the clearance of the pathogen [1,2]. However, microbial virulence factors may interfere with this clearance process thus resulting in acute or chronic infections or in some cases death of the host [3]. 

Innate immune cells express pattern recognition receptors called Toll-like receptors and NOD-like receptors that recognize microbial products such as lipopolysaccharides and peptidoglycans and allow them to mount an immune response, including production of inflammatory cytokines, chemokines and other antimicrobial agents such as the reactive oxygen and nitrogen species [4,5]. Lysosomal enzymes in phagocytic cells help degrade the pathogens, which are then presented to helper T cells via MHC class II molecules, and to cytotoxic T cells via MHC class I molecules. T helper cells produce cytokines that help B cells to respond by producing antibodies against specific pathogens while cytotoxic T cells can directly clear pathogens [6].

The host immune response and pathogen resistance may be influenced by the nutritional status in which dietary lipids, including fatty acids, play a major role as demonstrated by human and animal studies, and *ex vivo* and *in vitro* experiments [7]. Long-chain polyunsaturated fatty acids (PUFAs) are divided into two categories namely, omega-6 and omega-3, based on the location of the first double bond from the methyl end of the fatty acid molecule. Omega-6 or *n*-6 PUFAs have the first double bond between the 6th and 7th carbon atoms and the omega-3 or *n*-3 PUFAs have it between the 3rd and 4th fatty acids [8]. These PUFAs are considered essential since most mammals cannot synthesize these fatty acids and therefore have to acquire them from dietary sources. Omega-6 PUFAs have inflammatory properties mediated by increased arachidonic acid and prostaglandin E_2_ (PGE_2_) production [9,10], and omega-3 PUFAs are anti-inflammatory and immunosuppressive in nature [11]. These PUFAs could potentially alter the fate of intracellular bacterial burden based on their impact on the immune response, and therefore, fatty acids have to be properly titrated to avoid detrimental effects [12]. It has been speculated that these fatty acids induce changes in immune responses by altering membrane fluidity, lipid peroxide formation, eicosanoid production or gene regulation [13,14,15,16,17]. On the other hand, saturated fatty acids (SFAs), including short-chain fatty acids (SCFAs), have been shown to have either no effect or immune-enhancing/inflammatory effects, depending on the chain length [4,18,19]. Among SFAs, butyrate has been most studied for its effects on innate and adaptive immunity. For instance, butyrate has been shown to activate the innate immune response, stimulate antibody production upon immunization of broiler chickens, inhibit chemotaxis, increase expression of adhesion molecules and inflammatory cytokines in human colonic epithelial cells, umbilical vascular endothelial cells and leukocytes, and reduce nitric oxide production in macrophages [20,21,22,23,24]. These effects may be mediated by immune cell receptor activation and mobilization of intracellular calcium [25]. Additionally, butyrate has been demonstrated to inhibit proliferation of epithelial cells, macrophages and T lymphocytes, and induce caspase-3/7-mediated apoptosis of these cells [26,27]. Anti-inflammatory effects of butyrate include, but are not limited to, inhibition of functional differentiation of human dendritic cells [28], suppression of LPS-induced TNF-α release and NF-κB reporter activity in human neutrophils [29], and reduced inflammatory cytokine production in broiler chickens exposed to LPS [30].

Thus due to their effects on the immune system, some dietary fatty acids have been shown to influence pathogen clearance, including food-borne pathogens [31]. Food-borne illnesses are usually caused by improper handling, cooking, or storage of foods. The most common bacteria that cause food-borne illnesses include, but are not limited to, *Salmonella*, *E. coli*, *Campylobacter*, *Listeria*, *Shigella* and *Staphylococcus aureus*. In this review, we will summarize the current literature on the protective and/or detrimental effects of the most commonly consumed saturated and unsaturated fatty acids on food-borne bacterial infections.

## 2. Effect of Fatty Acids on *Salmonella* and *Campylobacter* Invasion, Colonization, and Clearance

Human salmonellosis is mainly caused by the consumption of raw or partially cooked eggs contaminated with *Salmonella enterica* serovars Enteritidis (SE) or Typhimurium (ST), which may also be transmitted by contaminated chicken meat. The global prevalence of *Salmonella* food poisoning has gone up significantly since 2001 [32], and this has caused a significant financial burden on the health care system [33]. The most common source of SE infection in chickens is contaminated feed in which SE is transmitted via infected mice and/or insects. Many micro and macronutrients are known to impact *Salmonella* infection in poultry as previously reported by us and others [34,35]. In addition to salmonellosis, poultry products are also known to be significant sources of human *Campylobacter* infections [36]. *Campylobacter* species including *C. jejuni* and *C. coli* are the most common bacterial causes of human gastroenteritis, with an estimate of more than 2 million cases per year in the US [37,38,39]. Medium-chain-length fatty acids (MCFAs) can mitigate *Campylobacter* in poultry. Below is a summary of the data reported up to date on the effects of various fatty acids on the clearance of *Salmonella* *enterica* serovars and *C. jejuni* in poultry and other species as well as in cell culture systems.

Among other nutrients, SCFAs have been used for decades as poultry feed additives due to their bactericidal properties. One of these properties is the ability to create an acidic environment in the intestinal tract, which is not favorable for bacterial growth [40]. Most of the studies conducted with SCFAs demonstrated increased *Salmonella* clearance from tissues and decreased shedding as shown in Table 1. Likewise, *in vitro* studies have demonstrated that SCFAs and MCFAs enhanced *Salmonella* clearance from various cells as shown in Table 2. Among the SCFAs, butyrate showed the most consistent antibacterial activity, which may be due to decreased invasion [41,42] via reduced expression of invasion genes [43] and increased induction of host defense peptides in the intestinal tract [44]. It was further demonstrated that the combination of SCFAs was more effective in mitigating *Salmonella* infection and inducing host defense peptides than if they were used individually [45].

**Table 1:** *In vivo* studies: Impact of dietary fatty acids on *Salmonella* control.

Species	Fatty acid	Measures: Organ colonization or mortality	Effect of Fatty acid on measures	Reference
Rhode Island red chickens	Dietary mixture of formic and propionic acids	*Salmonella gallinarum* strain 9 induced mortality	Decrease (↓)	[46]
Leghorn layer chickens	Dietary mixture of formic and propionic acids	Crop and cecal colonization with *Salmonella pullorum*	↓	[47]
Broiler chickens	Dietary butyric acid	*Salmonella* Enteritidis (SE) shedding in ceca. Crop, liver & spleen colonization	↓	[48]
Broiler chickens	Dietary caprylic acid	Ceca, crop, liver, small intestine, cloaca, liver & spleen colonization with SE	Dose dependent reduction	[49]
Young chicks	Dietary formic or propionic acid	Cecal colonization with *Salmonella* Typhimurium (ST)	↓	[50]
White leghorn chickens	Dietary formic, acetic, propionic or butyric acid	Cecal colonization with SE	↓ with butyric acid	[51]
Male broiler chicks	Dietary propionic acid	Crop and cecal colonization with ST	No difference (↔) with propionic acid	[52]
Lohmann white chicks	Dietary caproic acid	Cecal, hepatic and splenic colonization with SE	↓	[53]
Lohmann white chicks	Dietary butyric acid followed by intraesophageal SE infection	Shedding & cecal colonization with SE	↓	[54]
Male Cornish Rockbroiler chickens	Dietary butyric acid	Cecal colonization with SE	↓	[44]
Four day old male Cornish Rock broiler chickens	0.5% acetate, 0.2% propionate, or 0.1% butyrate individually or in combination	Cecal colonization with SE	↓	[45]
Pigs	Dietary lactic and formic acids	Shedding and sero prevalence	↓	[55]
Six week old piglets	Dietary butyrate, caprylate	Shedding and organ colonization	↔ with either fatty acid	[56]
Female Swiss and C57BL/6 mice	Intramuscular injection of liposome containing myristic, stearic or oleic acids	% survival after intraperitoneal (i.p.) infection with ST	Increase (↑) with myristic, stearic acid & oleic acid	[57]
Male Wistar rats	Dietary corn oil or fish oil (FO)	i.p. infection with SE	↔ in spleen and liver colonization with FO; ↓ in serum IFN-γ, delayed type hypersensitivity & IgG to *Salmonella* antigen in FO group	[58]

↓, Decrease; ↔, No difference; ↑, Increase.

**Table 2:** *In vitro* studies: Impact of dietary fatty acids on *Salmonella* invasion and clearance.

Cell model	Fatty acid	Measures: Invasion and clearance	Effect of fatty acid on measures	Reference
Study with avian intestinal cell line	Formic, acetic, propionic or butyric acid	SE invasion	↓ with butyric & propionic acids	[42]
Study with the chicken cecal epithelial cells	Acetic or butyric acid	SE invasion	↓ with butyric acid & ↑ with acetic acid	[41]
Study with chicken macrophage cell line	Arachidonic, α-linolenic, palmitic, stearic, linoleic, eicosapentanoic and docosahexanoic acids	SE clearance	↑ with α-linolenic & docosahexanoic acids	[59]
Study with chicken macrophage cell line (HD11), primary monocytes, bone marrow cells & jejunal, cecal explants	Butyric acid	Induction of host defense gene expression and SE clearance Oxidative burst, phagocytosis & macrophage activation	↑ ↔	[44]
Study with HD11 and primary monocytes	Butyrate, propionate, acetate individually or in combination; medium chain & long chain fatty acids	Induction of host defense peptide (HDP) gene expression	↑ HDP expression-Short chain fatty acids most effective (especially in combination), medium chain moderate; long chain fatty acids were marginal	[45]
Study with porcine intestinal epithelial cell line	Formic, acetic, propionic, butyric, caproic, caprylic, capric acids	ST invasion	↓ with propionic, butyric, caproic and caprylic acids	[57]

↓, Decrease; ↔, No difference; ↑, Increase.

The MCFA caprylic acid caused decreased tissue colonization in broilers, but had no impact on *Salmonella* shedding or organ colonization in piglets (Table 1, Table 2). The mechanism of action of caprylic acid may be similar to that of SCFAs in that MCFAs may inactivate bacteria by creating an acidic environment or by a direct impact on the expression of virulence factors necessary for *Salmonella* colonization. Few studies have examined the effect of fish oil PUFAs on *Salmonella* clearance and they are summarized in Table 1, Table 2. Fish oil PUFAs caused a general immunosuppression with no effect on *Salmonella* colonization in a rat study, although the bacteria were completely cleared in the liver and significantly reduced in spleens by 14 days post infection in all the dietary groups [58]. Furthermore, chicken macrophages pre-treated with α-linolenic and docosahexanoic acids showed increased *Salmonella* clearance with no change in superoxide or nitric oxide production (Table 1, Table 2). 

One of the most predominant nutritional intervention strategies among broilers to mitigate *Campylobacter* infection is inclusion of MCFAs. This is because of their known antibacterial activity against a wide range of microorganisms thus making them a great alternative to antibiotics [60]. Furthermore, MCFAs are generally recognized as safe (GRAS) by the Food and Drug Administration [61]. Most of the studies were conducted with broiler chickens, which are the main vehicle for food-borne campylobacteriosis, and have yielded conflicting data. For instance, day old broiler chicks that were fed a diet with 0.25% (w/w) caprylic and capric acids (1:1 ratio) or a mixture of capric, caprylic, and caproic acids showed significantly lower *Campylobacter* shedding and gastrointestinal tract colonization or lower incidence of cecal colonization [62,63]. Similarly, other studies conducted with day old chicks fed diets with different doses of caprylic acid alone have demonstrated significantly higher bacterial clearance in the ceca of birds that received a 0.7% or higher concentration of the fatty acid for last 3 or 7 days of the infection period [64,65,66]. However, two studies have shown that adding different doses of caprylic acid to drinking water or adding capric, caprylic or caproic acids to the feed for 3 days did not change the cecal colonization 11 days post infection in 70 or 27 day old broilers suggesting that the water soluble caprylic acid was absorbed in the intestine and did not reach ceca at levels adequate to clear the bacteria [67,68]. Among other SCFAs, butyrate has been shown to have antibacterial activity against *Campylobacter* in culture but it had no effect on cecal colonization when it was added to the broiler feed for two weeks prior to the oral challenge with *C. jejuni* [69,70]. These data indicate that MCFAs could offer a promising solution to alleviate human campylobacteriosis traced back to broilers.

## 3. Effect of Fatty Acids on Growth and Pathogenesis of Shiga Toxin-Producing *Escherichia coli* and *Shigella*

Human food-borne illness associated with Shiga toxin-producing *E. coli* (STEC) is mainly due to consumption of foods that have been contaminated with feces. While infections associated with *Shigella* occur mainly in developing countries with poor hygiene and unsafe water supplies, sporadic outbreaks occur in the United States through contaminated, uncooked food [71]. STEC and *Shigella* spp. can cause bloody diarrhea (hemorrhagic colitis and bacillary dysentery, respectively). STEC and *Shigella dysenteriae* type I produce potent cytotoxins known as Shiga toxins (Stxs) and can cause kidney complications (hemolytic uremic syndrome [HUS]) in susceptible individuals. While *S*. *dysenteriae* expresses the prototypical Stx, STEC produce Stx1 (essentially identical to Stx) and/or Stx2, which is 56% homologous to Stx/Stx1 [72]. Stxs are known to activate the ribotoxic stress response in host cells, which triggers signaling cascades that induce an innate immune response and cell death that ultimately lead to the progression of disease [73].

In the United States, the STEC serotype most associated with disease is O157:H7, and is acquired through the consumption of produce or undercooked beef products that have been contaminated [74]. However, non-O157 serotypes have emerged as a public health problem all over the world, primarily due to the globalization of the food supply [75]. The main reservoir of STEC is cattle [76], which makes the survival of STEC in cattle a primary concern. STEC survive asymptomatically in the recto-anal junction of cattle [77], and understanding the means of survival in cattle can lead to the possible reduction of bacteria that can contaminate food. Therefore, researchers have examined the *in vitro* and *in vivo* effects of fatty acids on STEC, including their ability to survive, grow, and colonize in cattle, as well as the effects of fatty acids on host immune responses to STEC (Table 3). The earliest research pertaining to the effects of fatty acids on *E. coli* O157:H7 compared bacterial growth in the ruminal environment of fasted animals to that of well-fed animals [78]. The ruminal environment of well-fed animals contains the SCFAs, acetate, propionate, and butyrate, which are weak acids with bactericidal properties at low pH [79]. As a result, this *in vitro* study showed that O157:H7 isolates grew poorly in media that simulated the ruminal environment of well-fed animals compared to that of fasted animals, suggesting that it is less likely for well-fed animals to become reservoirs. A few years later, another study demonstrated that combining plant metabolites and the SCFAs acetate, propionate, and butyrate inhibited *E. coli* O157 growth more than the individual components, suggesting that appropriate nutrition could help reduce the numbers of pathogenic *E. coli* in food animals prior to slaughter [80]. Nakanishi *et al.* [81] also found that high concentrations of a mixture of acetate, propionate, and butyrate inhibited growth of the O157:H7 *in vitro*, however, low concentrations enhanced the expression of virulence genes involved in adherence and pathogenesis. Specifically, butyrate had the greatest effect of enhancing the promoter activity of the locus for enterocyte effacement (LEE) 1 operon, which encodes the LEE encoded regulator (Ler), a global regulator of the LEE genes. These results suggest that SCFAs should be used with caution since they may enhance virulence of some O157:H7 strains. Despite these results, a recent study demonstrated that acetate produced by the protective *Bifidobacterium longum* subsp. *longum* was able to protect germ-free mice from lethal infection with *E. coli* O157:H7 possibly through anti-inflammatory and anti-apoptotic effects on colonic epithelia as well as blocking translocation of lethal doses of Stx2 [82].

**Table 3:** Impact of dietary fatty acids on Shiga toxin-producing *E. coli* growth and pathogenesis.

	Fatty Acid	Measures: Bacterial growth or host response	Effect of Fatty acid on measures	Reference
In vitro studies				
Bacterial culture	Acetate, propionate, & butyrate	O157:H7 933, 4477, 3081, & DBL No. 192-5-01, 336-2-02, 396-2-02, 647-6-04, & 768-2-01 growth	↓	[78]
Bacterial culture	Acetate, propionate, & butyrate	O157:H7 NCTC 12900 growth	↓	[80]
Bacterial culture	Acetate, propionate, & butyrate	O157:H7 Sakai growth	↓	[81]
	Butyrate	Virulence gene expression (Ler)	↑	
Human colonic epithelial cells Caco-2	Acetate	Translocation of Stx2	↓	[82]
Human blood monocytes & monocyte cell line U937	Arachidonic acid, or dihomolinolenic acid	Phagocytosis of unspecified, FITC-labelled O157:H7 strain	↑	[83]
		IL-1β production	↑	
Human renal tubular epithelial cell line ACHN	EPA, arachidonic acid, DHA, or α-linolenic acid	Cell death due to Stxs	↓	[84]
Bacterial culture	Bioconverted EPA or DHA	Unspecified human & ATCC 43888 O157:H7 strains growth	↓	[85]
Bacterial culture	Capric acid, lauric acid, or linoleic acid	CFUs of O157:H7 strain H4420N	↓	[86]
In vivo studies				
Mice	Acetate	Lethal infection with O157:H7 strain 44	↓	[82]
Cattle	Canola oil (oleic, linoleic, α-linoleic, & palmitic acids)	Shedding of O157:H7 strains E318N, R508N, E32511, & H4220N	↔	[87]

↓, Decrease; ↔, No difference; ↑, Increase.

In addition to SCFAs, the effects of MCFAs and PUFAs have also been examined for their ability to affect host response to STEC as well as STEC growth. For instance, arachidonic and dihomo-γ-linoleic acids were found to increase phagocytosis of fluorescein isothiocyanate (FITC)-labelled O157 and IL-1β production by monocytes [83]. In the renal epithelial tubule cell line ACHN, *n*-3 PUFAs appeared to decrease cell death caused by Stxs [eicosapentanoic acid (EPA) > (arachidonic acid (AA) = docosahexanoic acid (DHA) >> α-linolenic acid (LNA)], with EPA having the greatest effect [84]. A reduction in renal tubule pathology could be protective against the development of HUS. EPA and DHA, following microbial bioconversion, have also been shown to have antibacterial activity against *E. coli* O157:H7 as determined by inhibition zones and microbial inhibitory concentration [85]. Another *in vitro* study examined the effect of pH on the bactericidal activity of capric, lauric, palmitic, oleic, linoleic, and linolenic acids against *E. coli* O157:H7 [86]. As the pH decreased from 7.0 to 2.5, capric, lauric, and linoleic acids were able to significantly reduce O157:H7 colony-forming units (CFU), with capric and lauric acids having the greatest effect at the lowest concentrations, suggesting that inclusion of these fatty acids in cattle feed might reduce survival and colonization of O157:H7 in cattle. An earlier *in vivo* study supplemented corn- or barley-based feedlot diets with canola oil, which contains oleic (61%), linoleic (21%), α-linolenic (11%), and palmitic (4%) acids, but found no reduction of *E. coli* O157:H7 shedding by feedlot cattle, suggesting fatty acids were not able to affect O157:H7 survival and growth *in vivo* [87]. However, canola oil does not contain capric or lauric acids, which have been shown to have the greatest anti-*E coli* O157:H7 effect. Plus, the different strains of *E. coli* O157:H7 used in these studies may have different reactions to the various fatty acids. Further research is necessary to determine the beneficial effects of fatty acid supplementation in feedlot diets as well as on host immune responses against *E. coli* O157:H7.

*Shigella* infections are also a global public health problem, especially due to the emergence of multi-drug resistant *Shigella* species [71], requiring the development of alternative effective treatments and prevention strategies. SCFAs have been examined for their antimicrobial characteristics against *Shigella* beginning with *in vitro* experiments that looked at the inhibitory activity of formic and acetic acids on *Shigella flexneri* viability in culture (Table 4) [88]. Due to their antibacterial actions *in vitro*, SCFAs have also been evaluated for disease outcome *in vivo* (Table 4). For instance, Rabbani *et al*. found that adult rabbits intracolonically inoculated with *Shigella flexneri* 2a followed 24 h later with bolus infusions of a mixture of the SCFAs acetate, propionate, and n-butyrate every 6 h up to 120 h had improved outcomes of shigellosis [89]. Specifically, rabbits treated with the SCFA mixture had reduced fecal blood and mucus, improved clinical symptoms, and reduced mucosal congestion, cellular infiltration, necrosis, and numbers of *Shigella* in the colon. A few years later, butyrate was examined for its ability to improve disease outcome in an oral rabbit model of shigellosis [90]. Butyrate treatment resulted in reduced clinical illness, severity of colonic inflammation, and bacterial numbers in stools. Furthermore, the antimicrobial peptide CAP-18 was significantly up-regulated in surface epithelia in butyrate-treated rabbits, which was consistent with reports that its homologue, LL-37, is up-regulated in shigellosis patients [91].

**Table 4:** Impact of dietary fatty acids on *Shigella* viability and pathogenesis.

	Fatty Acid	Measures: Bacterial survival or clinical symptoms	Effect of Fatty acid on measures	Reference
*In vitro* study				
Bacterial culture	Formic or acetic acids	*Shigella flexneri* viability	**↓**	[88]
*In vivo* studies				
Adult rabbits	Acetate, propionate, & butyrate	After intracolonic *Shigella flexneri* 2a infection:		[89]
		fecal blood & mucus	**↓**	
		clinical symptoms	**↓**	
		mucosal congestion	**↓**	
		cellular infiltration	**↓**	
		necrosis	**↓**	
		*Shigella* in colon	**↓**	
Adult rabbits	Butyrate	After oral *Shigella flexneri* 2a infection:		[90]
		clinical illness	**↓**	
		colonic inflammation	**↓**	
		*Shigella* in stool	**↓**	
		Antimicrobial peptide CAP-18 in surface epithelium	**↑**	

↓, Decrease; ↔, No difference; ↑, Increase.

## 4. Effect of Fatty Acids on Colonization and Survival of *Listeria monocytogenes*

*Listeria monocytogenes* (*LM*) is a ubiquitous gram-positive food-borne pathogen, which causes serious disease especially in susceptible populations such as the immunocompromised or pregnant women. Several epidemiological studies have linked human listeriosis to specific foods, such as soft cheeses, melons or undercooked meat [92]. The clinical manifestations include but are not limited to gastroenteritis, meningitis and spontaneous miscarriage [93]. *Listeria* is known to survive at refrigerated temperatures and under other stress factors such as high pressure, which is used to inactivate microorganisms, thus making it difficult to eliminate this food-borne pathogen [94]. Murine listeriosis has been used as a model to study the impact of various dietary factors on disease outcome and its relationship to the immune response of the host. In this section we will briefly summarize the effect of fatty acids on listeriosis. Most of the studies involving long-chain PUFAs resulted in increased LM colonization, host mortality and intracellular survival of the bacteria (Table 5). These effects have been attributed to changes in immune cell populations and a general immunosuppression caused by these long-chain PUFAs [95,96,97,98]. On the other hand, a high milk fat diet, in which 40% of the calories were provided by butter oil and corn oil mixture (7:1 ratio), resulted in increased listericidal activity of gastric content and decreased fecal shedding of *Listeria* in rats. This bactericidal property of high milk fat was attributed to the increased SFAs with chain lengths varying from C4:0 to C18:0 in gastric contents of high milk fat fed rats [99].

**Table 5:** Impact of dietary fatty acids on *Listeria* *monocytogenes* colonization and survival.

Species	Fatty acid	Measures: Organ colonization or mortality	Effect of fatty acid on measures	Reference
8 week old BALB/c mice	Low fat, olive oil, fish oil or hydrogenated coconut oil (20% by weight) for 4 weeks	*Ex vivo* infection of peritoneal cells with LM at a multiplicity of infection (MOI) of 20:1	Fish oil (FO) caused ↑ bacterial survival within peritoneal cells compared to other lipids	[100]
*In vitro* treatment of peritoneal cells with 100 µM fatty acids	Oleic, stearic, eicosapentanoic, linoleic and linolenic acids	Bactericidal activity was measured 24 h post infection	Bacterial survival was ↑ with eicosapentanoic, linoleic and linolenic acids compared to control and other saturated fatty acids	[100]
8–10 week old BALB/c mice	Low fat, olive oil, fish oil or sunflower oil for 4 weeks	*Ex vivo* infection of spleen cells with LM at a MOI of 20:1	↑ LM mediated cytotoxicity of spleen cells by FO and olive oil; FO caused immunosuppression	[98]
8–10 week old BALB/c mice	Low fat, olive oil, fish oil or hydrogenated coconut oil (20% by weight) for 4 weeks	10^5^ LM through tail vein	↓ survival and increased liver and spleen colonization in FO group	[101]
8–10 week old BALB/c mice	Low fat, olive oil, fish oil or hydrogenated coconut oil (20% by weight) for 4 weeks	10^4^ LM through tail vein	↑ spleen colonization in FO group	[102]
8–10 week old BALB/c mice	Low fat, olive oil, fish oil or hydrogenated coconut oil (20% by weight) for 4 weeks	10^4^ LM through tail vein	↑ spleen colonization in FO group at 24, 48, 72 and 96 h post infection (PI) and in hydrogenated coconut oil group at 96 h PI	[103]
8–10 week old BALB/c mice	Low fat, olive oil, fish oil or hydrogenated coconut oil (20% by weight) for 4 weeks	*Ex vivo* infection of thymocytes with LM at a MOI of 20:1	No effect on cytoxicity by any of the dietary fatty acid	[104]
10 week old BALB/cmice	Low fat, olive oil, fish oil or hydrogenated coconut oil (20% by weight) for 4 weeks	10^4^ LM through tail vein	↓ survival and ↑ spleen colonization in all oil groups compared to low fat group	[7]
8 week old BALB/c mice	Low fat, olive oil, fish oil or sunflower oil for 8 weeks	*In vivo* infection with primary-10^3^ LM and secondary-10^4^ LM for colonization and 10^5^ LM for survival studies 28 days after primary injection through tail vein	100% survival in the FO group; spleen colonization ↓ at 72 h compared to 24 h in FO group	[105]
3–4 week old BALB/cAnNHsd mice	Lard or fish oil diet for 4 weeks	*In vivo* infection with 2 × 10^5^ LM intravenously	↑ spleen and liver colonization in the FO compared to the lard group	[106]
3 week old C3H/HeN mice	Lard, soybean or fish oil diet for 4 weeks	i.p. infection with 2 × 10^6^ LM	Survival 100%, 58% and 33% for lard, soybean or fish oil, respectively ↑ spleen colonization in FO group	[107]
3–4 week old BALB/cAnNHsd mice	Lard or fish oil diet for 4 weeks	Intravenous infection (i.v.) with 1.4 × 10^4^ LM	↑ spleen and liver colonization in the FO compared to the lard group	[108]
3–4 week old BALB/cAnNHsd mice	Lard or fish oil diet for 4 weeks	i.v. infection with 10^5^ or 10^6^ LM	↓ survival of mice in FO compared to lard group (100% at 10^5^ dose and 30% at 10^6^ dose) by day 14; ↑ spleen and liver colonization in FO compared to lard group	[96]
6 week old female CD1 mice	Conjugated linoleic acid or control diet for 14 or 32 days	i.p. LM 2.5 × 10^5^ or 1.5 × 10^5^ in the two experiments, respectively	↔ spleen and liver colonization or histopathological changes due to LM infection	[109]
9 week old male Wistar rat	Rats were fed 10% or 40% fat diets corresponding to 4.2% & 19.6% milk fat for 2 weeks. *In vitro* study with different fatty acids in milk up to 2 mM	Oral infection by gastric gavage with 5 × 10^9^ LM *in vitro* experiments done with 10^8^ LM for 2 h	High milk fat diet ↓ fecal LM excretion, ↑ listericidal activity of gastric contents listericidal activity of fatty acids ranked in the order C14:0 < C18:2 < C10:0 < C18:1 < C12:0	[99]

↓, Decrease; ↔, No difference; ↑, Increase.

## 5. Fatty Acids and *Staphylococcus aureus*

*Staphylococcus aureus* is a facultative anaerobic Gram-positive bacterium that causes gastroenteritis, and food poisoning usually results from ingestion of a heat stable toxin produced by the bacteria. *S.* *aureus* is generally found in the nostrils, skin and hair of warm-blooded animals and 30%–50% of humans are known to be carriers of this pathogen [110]. The symptoms of staphylococcal food poisoning include abdominal cramps, nausea, vomiting, and diarrhea. *S.* *aureus* outbreaks are attributed to a variety of foods including beef, pork, milk and cheese prepared from raw milk produced by cows suffering from mastitis, and cheese from food handlers who are carriers of *S.* *aureus* or those that follow poor hygiene practices [111,112]. The presence of methicillin-resistant *S.* *aureus* (MRSA) in contaminated foods has been reported in recent years [113,114], although it is mostly associated with nosocomial staphylococcal infections, which cause worldwide morbidity and mortality [115]. Whether or not they are methicillin-resistant, food-borne *S.* *aureus* can pose a serious public health problem and economic burden throughout the world [116,117]. Several dietary intervention strategies have been tested for decades to arrive at effective antimicrobial measures against *S.* *aureus*, including MRSA. These include but are not limited to tea and coffee consumption associated with lower incidence of MRSA nasal carriage (as a population survey) [118] and dietary glutamine being effective in reducing the mortality rate in BALB/c mice challenged with MRSA [119]. Furthermore, human and animal skin, breast milk, and blood naturally contain free fatty acids, which have antibacterial activity, thus making them an obvious choice for experimental and clinical intervention studies. Below is a summary of studies related to the effects of fatty acids on *S.* *aureus* infection in various animal models, cell cultures and on pathogen virulence factors. 

Consumption of a high fat diet resulted in increased mortality in mice infected with *S.* *aureus*, which was associated with suppression of innate immune responses [120]. However, studies with fish oil have yielded contradicting data in that rabbits fed high fish oil and safflower oil showed reduced bacterial clearance [121], while pigs fed fish oil prior to surgical insertion of an aortic vascular prosthetic graft showed increased body weight gain compared to those fed sunflower oil, with no change in clinical signs of infection [122]. It is likely that the newborn rabbits were more sensitive to dietary PUFAs, which may cause a general immunosuppression, while weight gain in pigs given fish oil was attributed to lower PGE_2_ levels. Other essential oils such as monolaurin and origanum oil have proven to reduce mortality in mice infected with *S.* *aureus*, either individually or in combination [123], which makes these natural fatty acids a good alternative or supplement to pharmaceuticals in fighting infections. In addition to these *in vivo* studies with different animal models, several *in vitro* studies demonstrated that most of the free fatty acids had bactericidal activity against *S.* *aureus* species as summarized in Table 6. These free fatty acids are naturally present in bovine and human milk and are increased during mastitis in cows, which suggests bactericidal effects.

**Table 6:** Impact of dietary fatty acids on *Staphylococcus aureus* infection.

Animal species/cell culture	Fatty Acid	Measures: Organ Colonization or mortality	Effect of Fatty acid on measures	Reference
Cystic fibrosis (CF) patients	Correlating essential fatty acid deficiency to respiratory disease	Increased susceptibility of CF patients to *S.* *aureus* infections	Plasma phospholipid fatty acids revealed that all CF patients had ↓ *n*-3 and *n*-6 fatty acids	[124]
5–7 week old male C57BL/6 or Ob/Ob mice	Low (4%) *versus* high (36%) fat diet for 8 weeks	5 × 10^7^ cfu intravenous injection in the tail vein	↓ survival, 10 fold higher bacteria in kidneys, ↑ serum IL-1β, ↓ reactive oxygen species by peritoneal cells in high fat group	[120]
One day old New Zealand white rabbits	High (5 g/kg body weight [bw]) or low (0.22 g/kg bw) fish oil or safflower oil for 8 days	30 min exposure to *S.* *aureus* aerosol to produce intrapulmonary infection	↓ bacterial clearance in high fish and safflower oil groups	[121]
28 day old pigs	10% fish oil, sunflower oil or animal fat for 35 days	After 3 weeks of dietary treatment, pigs had aortic vascular prosthetic graft inserted which was inoculated with 10^6^ cfu *S. aureus* and monitored for 14 days	↔ in clinical signs of infection such as rectal temperature, hindquarter function, general appearance and feed intake ↑ body weight gain in FO compared to sunflower oil group	[122]
5–7 week old BALB/c mice for *in vivo* study and *in vitro* addition of fatty acids to bacterial cultures	Daily gavage with origanum oil, monolaurin or the combination in 0.2 mL olive oil for 30 days	Injected with 5 × LD_50_ *S.* *aureus* ATCC 14775; susceptibility tested as minimum inhibitory and minimum bactericidal concentrations [MBC] (ATCC 14154 & 14775)	4/8 mice survived in the monolaurin group at 30 days & 5/8 survived in combination group; monolaurin & origanum oils were most potent against *S.* *aureus* ATCC 14154 & 14775	[123]
*In vitro* addition of fatty acids to bacterial cultures	Final concentrations of fatty acids were 0, 12.5, 25, 50, 100 or 200 μg/mL	3 *S.* *aureus* strains were used (*S.* *aureus* MN8 (human isolate) *S.* *aureus* Novel and 305 (clinical bovine mastitis isolates)), and incubated with fatty acids for 24 h	7 most potent inhibitors were lauric acid, glycerol monolaurate, capric, myristic, linoleic & conjugated linoleic acids; lauric, capric and myristic acids reduced overall growth; linoleic and conjugated linoleic acids delayed the initiation of exponential growth	[125]
*In vitro* addition of fatty acids to bacterial cultures	0, 0.25, 0.5 & 1 mM linoleic acid	4 wild type *S.* *aureus* strains-SH1000, MRSA252, MSSA476 & N315	↓ survival of all 3 strains of *S.* *aureus* by linoleic acid especially at 1mM concentration	[126]
*In vitro* addition of fatty acid to assess MBC using pork loin	Lauric acid, monolaurin and lactic acid, virgin coconut oil	2 strains of *S.* *aureus*-ATCC 25923 and an isolate from pig carcass	↓ bacterial counts with lauric acid, monolaurin and lactic acid; ↔ with virgin coconut oil	[127]
*In vitro* addition of fatty acids to bacterial cultures	Lauric acid, d-sphingosine, phytoshpingosine, dihydro-sphingosine & sapienic acid	*S.* *aureus* ATCC 29213	All lipids were bactericidal, except sapienic acid	[128]
*In vitro* addition of sugar fatty acid esters to bacterial cultures	Sugar fatty acid esters with (C8–C16)	*S.* *aureus* A7510	Fatty acids C10–C16 ↓ biofilm formation; C14 and C16 were bactericidal	[129]
*In vitro* addition of fatty acids to bacterial cultures	Capric (20ppm), lauric & α-linolenic acids (1 ppm)	*S.* *aureus* ATCC 13565	↓ bacterial growth with lauric & α-linolenic acids but ↔ with capric acid	[130]

↓, Decrease; ↔, No difference; ↑, Increase.

## 6. Conclusions

Overall, fatty acids have diverse roles in the way they affect the immune system and bacterial clearance and no single dietary fatty acid is suitable for treating all food-borne pathogens. For *Salmonella* mitigation in chickens, short-chain fatty acids may offer a potential intervention strategy, but for *Campylobacter* medium-chain fatty acids could be more effective. Shiga toxin-producing *E. coli* growth and pathogenesis appear to be affected by short-chain, medium-chain and polyunsaturated fatty acids, requiring further research to determine the best intervention and treatment methods, while *Shigella* appear to be susceptible to only short-chain fatty acids. There is no clear fatty acid choice for *Listeria monocytogenes* clearance, however fish oil may have detrimental effects on the immune response and *Listeria monocytogenes* burden. With respect to *Staphylococcus* *aureus* clearance, fish oil showed contradicting effects, arachidonic acid was detrimental, but oleic and lauric acids appeared beneficial, albeit there are limited studies to confirm these effects. Although PUFAs may be beneficial for reducing inflammatory cardiovascular diseases and preventing bone loss, they may be immunosuppressive and therefore may result in reduced host resistance to certain bacterial infections. It is important to conduct more large scale studies with relevant animal models to arrive at meaningful recommendations for various fatty acid interventions for clinical settings or the improved health of animals used for human consumption.
